# COMP and TSP-4: Functional Roles in Articular Cartilage and Relevance in Osteoarthritis

**DOI:** 10.3390/ijms22052242

**Published:** 2021-02-24

**Authors:** Kathrin Maly, Enrique Andres Sastre, Eric Farrell, Andrea Meurer, Frank Zaucke

**Affiliations:** 1 Research Unit for Osteoarthritis, Department of Orthopaedics (Friedrichsheim), University Hospital Frankfurt, Goethe University, Marienburgstraße 2, 60528 Frankfurt/Main, Germany; kathrin_maly@gmx.at (K.M.); andrea.meurer@kgu.de (A.M.); 2 Department of Oral and Maxillofacial Surgery, Erasmus MC, University Medical Centre Rotterdam, Wytemaweg 80, 3015 CN Rotterdam, The Netherlands; enadestre2@gmail.com (E.A.S.); e.farrell@erasmusmc.nl (E.F.)

**Keywords:** COMP, TSP-4, extracellular matrix, articular cartilage, osteoarthritis

## Abstract

Osteoarthritis (OA) is a slow-progressing joint disease, leading to the degradation and remodeling of the cartilage extracellular matrix (ECM). The usually quiescent chondrocytes become reactivated and accumulate in cell clusters, become hypertrophic, and intensively produce not only degrading enzymes, but also ECM proteins, like the cartilage oligomeric matrix protein (COMP) and thrombospondin-4 (TSP-4). To date, the functional roles of these newly synthesized proteins in articular cartilage are still elusive. Therefore, we analyzed the involvement of both proteins in OA specific processes in in vitro studies, using porcine chondrocytes, isolated from femoral condyles. The effect of COMP and TSP-4 on chondrocyte migration was investigated in transwell assays and their potential to modulate the chondrocyte phenotype, protein synthesis and matrix formation by immunofluorescence staining and immunoblot. Our results demonstrate that COMP could attract chondrocytes and may contribute to a repopulation of damaged cartilage areas, while TSP-4 did not affect this process. In contrast, both proteins similarly promoted the synthesis and matrix formation of collagen II, IX, XII and proteoglycans, but inhibited that of collagen I and X, resulting in a stabilized chondrocyte phenotype. These data suggest that COMP and TSP-4 activate mechanisms to protect and repair the ECM in articular cartilage.

## 1. Introduction

Articular cartilage consists of an extracellular matrix (ECM) with a unique composition and architecture, providing the biomechanical properties for frictionless motion [1,2,3]. The tissue is organized in four zones: the superficial zone at the top, the transitional zone, the deep zone and the calcified zone [1]. Also, the area around the chondrocytes is subdivided into a pericellular matrix directly surrounding the chondrocytes, a territorial matrix, surrounding the pericellular matrix and the interterritorial matrix, the area between the territorial matrices. The main components of the ECM are proteoglycans and collagens, responsible for elasticity and stiffness, respectively [1]. The predominant collagen II forms fibrils with collagen XI as a core and collagen IX on its surface [4,5,6]. The distribution of different collagen types depends on the zonal localization. Thinner collagen fibrils are associated with collagen IX [5,6], while thicker fibrils are not [6,7]. Fibrils in the superficial zone are also associated with collagen XII [8]. Collagen IX and XII both belong to the fibril-associated collagens with interrupted triple helices (FACIT). They are structurally similar [9] but, in contrast to collagen IX, collagen XII is noncovalently linked to fibrillar collagens [10]. Collagen X is restricted to the calcified zone and represents a transitional area, linking the articular cartilage to the bone [11,12,13,14]. To a minor extent, the ECM also contains noncollagenous proteins [2], e.g., the cartilage oligomeric matrix protein (COMP), which promotes collagen secretion and assembly as well as ECM stability [15,16]. Chondrocytes are surrounded by a dense ECM and remain quiescent in mature cartilage, mediating ECM homeostasis in a low turnover state [17]. In osteoarthritis (OA), alterations at the cellular and molecular levels cause a continuous degradation of the ECM, leading to joint failure and pain [18,19]. Chondrocytes produce degrading proteases and are reactivated to synthesize ECM proteins, resulting in ECM remodeling [15,18,20,21,22]. The cause of chondrocyte reactivation is not completely understood, but might be regulated by cell-matrix interactions. Growth factors like TGF-β1 [23,24,25,26], which are commonly stored in the ECM and released under certain circumstances, play an important role by regulating processes like cell proliferation and differentiation [27,28], in particular via the Smad and Erk signaling pathways [24,29,30,31,32]. The level of activated TGF-β1 is increased in the synovial fluid of OA patients [33], suggesting enhanced interaction with chondrocytes in cases of disease. During OA progression, the phenotype of chondrocytes becomes unstable [34], resulting in the loss of chondrogenic markers (collagen II, collagen IX and COMP) [35] and the synthesis of ECM components that are usually not expressed in articular cartilage, like collagen I and collagen X [36,37,38]. The ECM protein COMP is a target of degradation in early OA, but is also re-expressed in later stages [15]. In contrast, its close family member, thrombospondin-4 (TSP-4), is hardly detectable in healthy cartilage, but its expression increases dramatically in OA cartilage and correlates with disease severity [39]. Both proteins have similar structures and binding partners [40,41,42,43,44,45,46], suggesting similar or additive roles in articular cartilage. It has also been reported that COMP binds TGF-β1, thereby promoting its transcriptional capacity [47], while this has not yet been shown for TSP-4. The re-expression of COMP and the new synthesis of TSP-4 in OA was interpreted as an attempt to restore the integrity of the cartilage ECM [15,39]. However, the effect of these ECM proteins on the chondrocyte phenotype and behavior, as well as their contribution to OA relevant processes and ECM repair, have not yet been investigated in detail. Therefore, the present study focuses on the potential of COMP and TSP-4 to promote repopulation of damaged cartilage areas by attracting chondrocytes, stabilizing their phenotype as well as inducing the synthesis and deposition of collagens and proteoglycans. The obtained data will contribute to a better understanding of the functional role of COMP and TSP-4 in articular cartilage, and might help to improve and develop effective therapeutic applications.

## 2. Results

### 2.1. COMP and TSP-4 Are Differentially Distributed in Human Healthy and OA Knee Articular Cartilage

**Figure 1:**
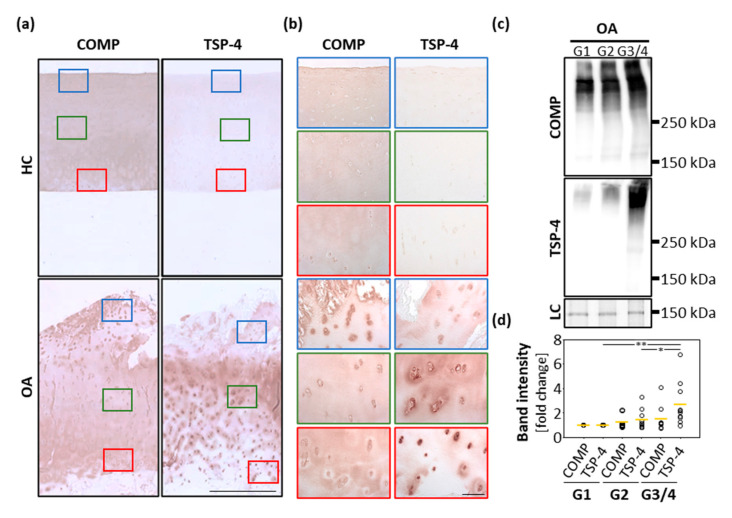
Localization and expression of COMP and TSP-4 in human articular cartilage. (**a**) Articular cartilage sections from femoral condyles from healthy controls (HC) and OA patients (OA) were stained with COMP and TSP-4 specific antibodies. Scale bar = 1 mm. (**b**) Magnifications of tissue areas indicated with the boxes in (**a**). Scale bar = 100 µm. (**c**) Total COMP and TSP-4 content was investigated via immunoblots, with proteins extracted from OA cartilage areas showing different degeneration grades (grade 1 (G1), grade 2 (G2) and grade 3/4). PageBlue^TM^ staining was used as loading control (LC). Representative immunoblots from 10 different donors are shown. (**d**) Quantification and statistical analyses of the immunoblots. Values are represented as scatter plots with indicated mean as fold changes to G1 (set as 1). The significance of the increase in COMP and TSP-4 is indicated (*p* < 0.05 *, *p* < 0.01 **).

Healthy and osteoarthritic cartilage tissue was paraffin-embedded and sections were stained for COMP and TSP-4 (Figure 1a,b). In healthy cartilage, COMP was ubiquitously and uniformly expressed throughout all layers. In the deep zone, the staining was mainly found in the interterritorial matrix. In addition, faint intracellular staining was observed. In OA cartilage, the staining in the superficial zone became weaker, and interterritorial COMP seemed primarily to be degraded. Instead, a pronounced intracellular staining of COMP was detected in the superficial zone, suggesting a re-expression of the protein. TSP-4 was hardly detectable in healthy cartilage, but could be detected in high levels in OA cartilage. While COMP was predominantly re-expressed at the cartilage surface, increased expression of TSP-4 was found mainly in the middle and deeper zone of articular cartilage.

In order to get a deeper insight into the amounts and structural integrity of COMP and TSP-4 in OA cartilage, total protein extracts from areas with different degradation stages were generated (grade 1 (G1): smooth surface and no fissures; grade 2 (G2): superficial discontinuities and fissures and grade 3/4 (G3/4): deep fissures and visibility of the subchondral bone). An immunoblot analysis of these extracts (Figure 1c) revealed that in the tissue TSP-4 was mainly present as a full-length pentameric protein, while COMP already showed signs of degradation, indicated by bands of lower molecular weight. Further, the total amount of COMP and TSP-4 was evaluated and the fold change in G2 and G3/4 samples related to G1 (set as 1) was calculated. The total amount of both proteins increased with disease severity. Interestingly, the increase in protein levels was much more pronounced for TSP-4 than for COMP. This increase was only significant for TSP-4 (Figure 1d).

### 2.2. COMP but Not TSP-4 Promotes Chondrocyte Migration and Attachment

**Figure 2:**
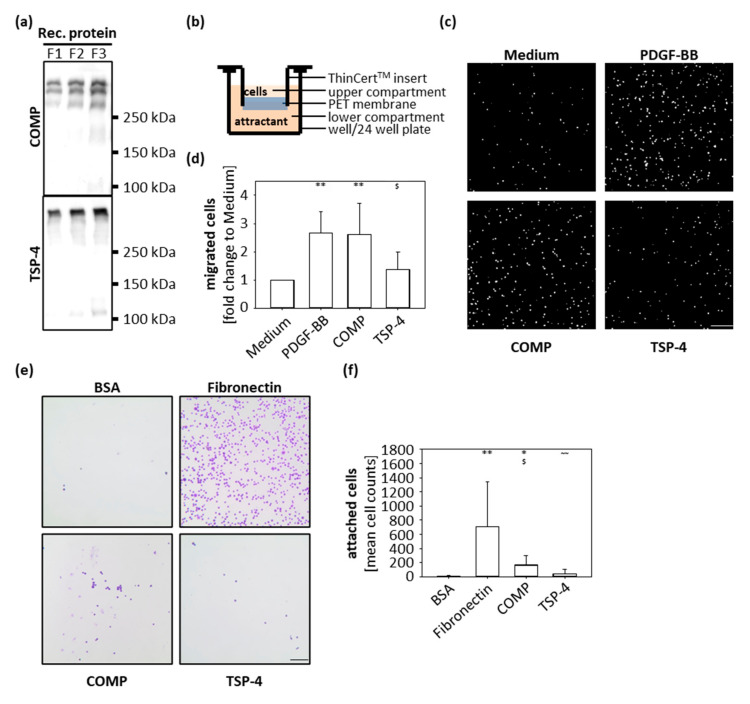
Chondrocyte migration and attachment to COMP and TSP-4. (**a**) Recombinant (rec) COMP and TSP-4 were eluted from the columns in several steps and every protein fraction (F) analyzed by immunoblot. (**b**) Schematic illustration of the transwell system. Porcine chondrocytes were added to the upper compartment and allowed to migrate towards COMP (10 µg/mL) or TSP-4 (10 µg/mL) for 10 h. (**c**) Representative images of migrated chondrocytes stained with DAPI. (**d**) Migrated cells were counted and cell numbers statistically evaluated. Each bar shows the mean + SD and significance (to medium: *p* < 0.01 ** and to PDGF-BB: *p* < 0.05 $) was analyzed. (**e**) Representative images of attached chondrocytes stained with crystal-violet. (**f**) Attached cells were counted and cell numbers statistically evaluated. Each bar shows the mean + SD and significance (to BSA *p* < 0.05 *, *p* < 0.01 **, to fibronectin *p* < 0.05 $ and COMP *p* < 0.01 ~~) was analyzed. The standard medium was used as a negative and PDGF-BB (10 ng/mL) as a positive control for the migration assay. BSA (100 mg/mL) was used as a negative control and fibronectin (10 µg/mL) as a positive control for cell attachment. (*n* = 4–5); scale bar = 100 µm.

In order to analyze the function of COMP and TSP-4 during OA progression, recombinant proteins were expressed and purified for downstream in vitro assays (Figure 2a). Both recombinant proteins could be produced in sufficient amounts and showed a high structural similarity to the naturally occurring proteins in OA cartilage (Figure 1c).

The immunohistochemical staining showed that the localization of COMP and TSP-4 differs between healthy and diseased cartilage; while COMP is re-expressed in superficial layers, TSP-4 is predominantly expressed in the middle and deep zones in OA conditions. To gain knowledge about the capacity of COMP to attract chondrocytes and contribute to their migration into the areas of damage to support repair undertakings, transwell migration assays were performed. As attractants, COMP (10 µg/mL) and TSP-4 (10 µg/mL) were added to the lower compartment, and porcine chondrocytes in the upper compartment were allowed to migrate for 10 h (Figure 2b). PDGF-BB (10 ng/mL) was used as a positive and standard medium as a negative control. Both COMP (*p* = 0.014) and PDGF-BB (*p* = 0.012) attracted chondrocytes compared to the negative control (Figure 2c,d). The number of cells migrating towards TSP-4 was similar to the negative control. (Figure 2c,d).

To determine whether COMP could also facilitate the attachment and anchorage of the attracted chondrocytes, wells of a 96-well plate were coated with 10 µg/mL COMP and also TSP-4; 10 µg/mL fibronectin was used as a positive control and 100 mg/mL BSA as a negative control. Chondrocytes were incubated in coated wells for 1 h, the attached cells were stained with crystal-violet and analyzed by light microscopy (Figure 2e,f). Chondrocytes attached to fibronectin (*p* = 0.003) and COMP (*p* = 0.012) but not to TSP-4 (*p* = 0.18). Also, an increased attachment for COMP compared to TSP-4 (*p* = 0.007) was observed. These data demonstrated that COMP but not TSP-4 has the potential to attract chondrocytes and facilitate their attachment.

### 2.3. Both COMP and TSP-4 Modulate Collagen and Proteoglycan Synthesis

**Figure 3:**
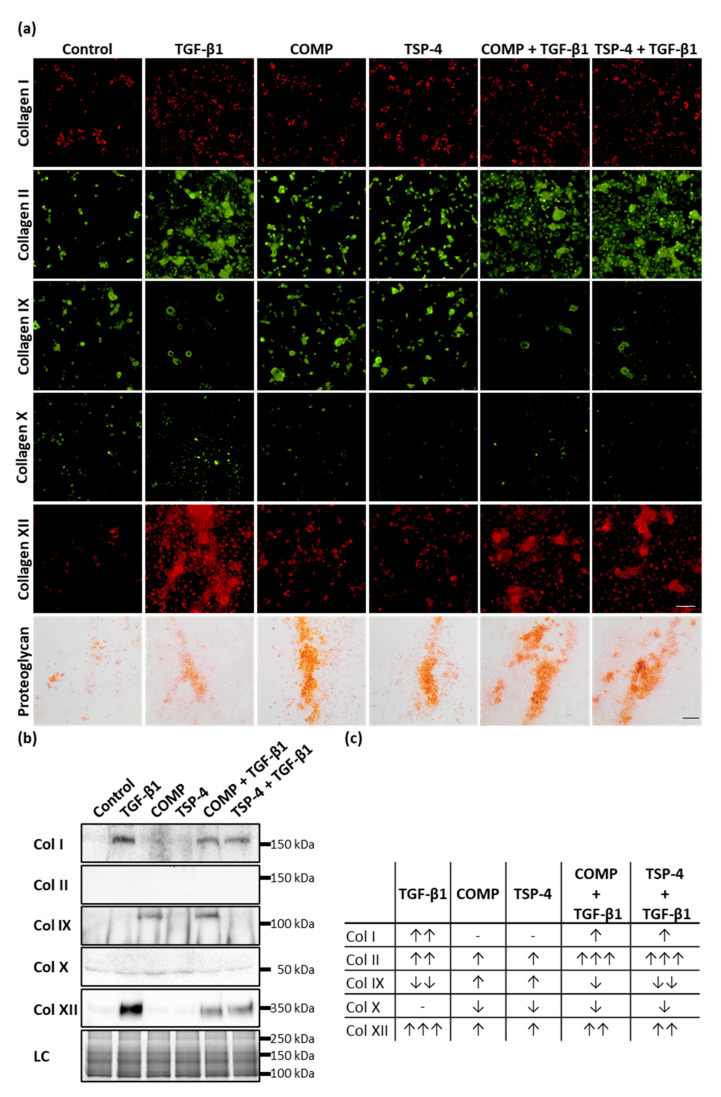
The effect of COMP and TSP-4 on collagen and proteoglycan production and *deposition.* Chondrocytes were stimulated with COMP (10 µg/mL), TSP-4 (10 µg/mL) alone or in combination with TGF-β1 (0.5 ng/mL) for seven days. (**a**) Immunofluorescence staining of collagen (Col) I, II, IX, X and XII as well as Safranin-O staining of proteoglycans at day 10. (**b**) Immunoblot assays of chondrocyte supernatants at day 10. Roti^®^blue staining of proteins was used as a loading control (LC). (**c**) Table summarizing the effect of COMP, TSP-4 and TGF-β1 on the total amount of different collagen types (matrix-associated and soluble). The amount of matrix-associated collagens was visually scored from IF staining and combined with band intensities for soluble proteins derived from immunoblots. The number of arrows indicates a strong, moderate or mild effect compared to the control. The direction of the arrows indicates an increase (↑) or decrease (↓) compared to control. No changes are indicated by a minus. The number of arrows indicates strong (three arrows), moderate (two arrows) or mild (one arrow) changes compared to control. (Immunofluorescence staining, *n* = 3; immunoblot *n* ≥ 2 per group); unstimulated cells were used as a control; scale bar (collagens) = 100 µm; and (proteoglycans) = 200 µm.

**Figure A1:**
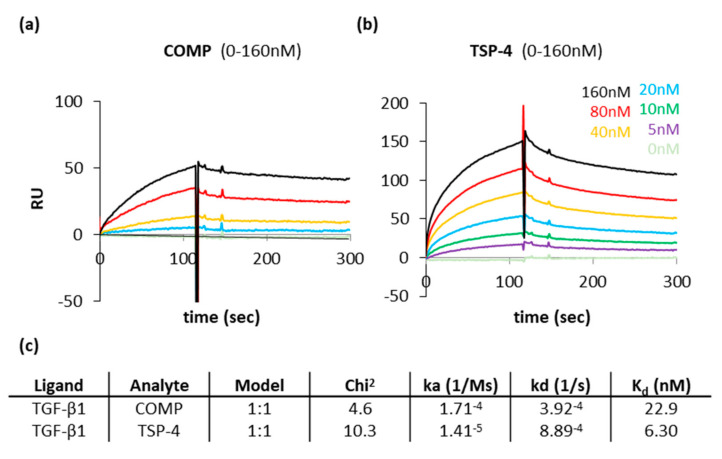
Surface plasmon resonance measurements show a direct interaction of COMP and TSP-4 with TGF-β1. Protein-protein interaction profiles showing the binding of COMP and TSP-4 to TGF-β1. Surface plasmon resonance sensorgrams generated with the Biacore system are shown for COMP (**a**) and TSP-4 (**b**). Resonance signals expressed as response units (RU), indicate the degree of binding COMP and TSP-4 with TGF-β1. COMP and TSP-4 were used in concentrations ranging from 5 to 160 nM and injected to immobilized TGF-β1 on a CM5 chip. (**c**) Summary of the data received from Biacore assays. The association rate (ka), the dissociation rate (kd), the dissociation constant (K_d_) and the chi^2^ are represented. A kinetic 1:1 model (Langmuir model) was used to calculate these parameters.

To determine whether COMP and TSP-4 could further stimulate chondrocytes to synthesize collagens and proteoglycans to rebuild the ECM in damaged areas and contribute to ECM remodeling in lower cartilage areas, chondrocytes were stimulated with COMP (10 µg/mL), TSP-4 (10 µg/mL), TGF-β1 (0.5 ng/mL) or in combination of TGF-β1 with COMP or TSP-4 for seven days. To ensure that the chondrocyte phenotype in monolayer culture remained stable, the stimulation experiments were stopped after three more days. COMP and TSP-4 directly interact with TGF-β1 (Appendix A, Figure A1), and COMP is further known to modulate the transcriptional activity of TGF-β1 [47]. Therefore, TGF-β1 alone was used as an inducer of collagen synthesis (positive control), while the combination with COMP and TSP-4 was used to observe their potential to modulate TGF-β1-induced effects. As a negative control, chondrocytes were cultured in standard medium. Matrix-associated collagens were detected by immunofluorescence staining, and matrix-associated proteoglycans were stained with Safranin-O (Figure 3a).

**Figure A2:**
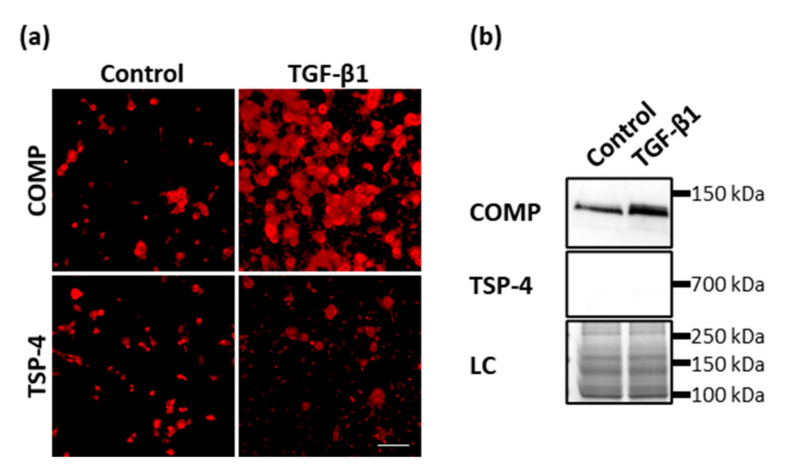
The effect of TGF-β1 on COMP and TSP-4 synthesis and deposition. Chondrocytes were stimulated with TGF-β1 (0.5 ng/mL) for seven days. COMP and TSP-4 were detected with specific antibodies. (**a**) Immunofluorescence staining of COMP and TSP-4 at day 10. (**b**) Immunoblot assays of chondrocyte supernatants at day 10. Roti^®^blue staining of proteins was used as a loading control (LC). (*n* = 3 per group); unstimulated cells were used as a control; scale bar = 100 µm.

COMP and TSP-4 stimulation resulted in an increased staining intensity of matrix-associated collagen II, IX, XII and proteoglycan, while no effect on collagen I and a slight decrease of collagen X deposition could be observed. TGF-β1 enhanced the matrix formation of collagen I, II, XII and proteoglycan while reducing collagen IX levels and not affecting collagen X. Investigating the effect of COMP and TSP-4 on TGF-β1 induced matrix formation showed a decrease of collagen I, X and XII levels, as well as an increase of collagen II and proteoglycan. TGF-β1 associated collagen IX reduction could not be reversed by COMP or TSP-4. By investigating the effect of TGF-β1 on the expression of COMP and TSP-4, we showed that TGF-β1 only induces the synthesis of COMP, but not of TSP-4 (Appendix B, Figure A2).

Collagens that are not integrated into the matrix remain soluble and can be detected in the cell culture supernatant. Therefore, the amount of different collagen types was analyzed by immunoblot assays (Figure 3b). Soluble collagen IX could only be detected in supernatants of cells treated with COMP, either alone or in combination with TGF-β1. This result showed that COMP contributes to the synthesis of collagen IX, even though its deposition into the matrix seems not to be significantly increased. Elevated amounts of soluble collagen I and XII could be observed only in TGF-β1 stimulated cells; this upregulation parallels the increase of the respective proteins in the matrix. Interestingly, the amount of the same proteins in the supernatant could be reduced by the addition of COMP or TSP-4. The signals for collagen X and collagen II in the supernatant were either very weak or absent altogether, indicating that these proteins are quantitatively incorporated into the cell-associated matrix.

Total levels of collagens (matrix-associated and soluble), detected by immunofluorescence staining and immunoblots (Figure 3c), showed that COMP and TSP-4 stimulation increased the total level of collagen II, IX and XII, and decreased that of collagen X. TGF-β1 increased the expression of collagen I, II and XII while reducing that of collagen IX. These effects on collagen I and XII were weakened by the addition of COMP and TSP-4, while the increase of collagen II expression was further enhanced.

### 2.4. COMP and TSP-4 Do Not Affect Proliferation but Suppress Dedifferentiation of Chondrocytes

Reactivated chondrocytes in OA proliferate and dedifferentiate to collagen I producing cells. COMP and TSP-4 affect collagen II matrix deposition and suppress TGF-β1-induced collagen I expression. The primary aim of the following experiments was not to study the dedifferentiation process itself, but to determine whether COMP and TSP-4 could directly or indirectly modulate this process. Chondrocytes were stained as described in Section 2.3 and studied in more detail by evaluating their proliferation and number of total, as well as the ratio, of collagen I and collagen II positive cells. These experiments were repeated three times with different batches of freshly isolated chondrocytes.

**Figure 4:**
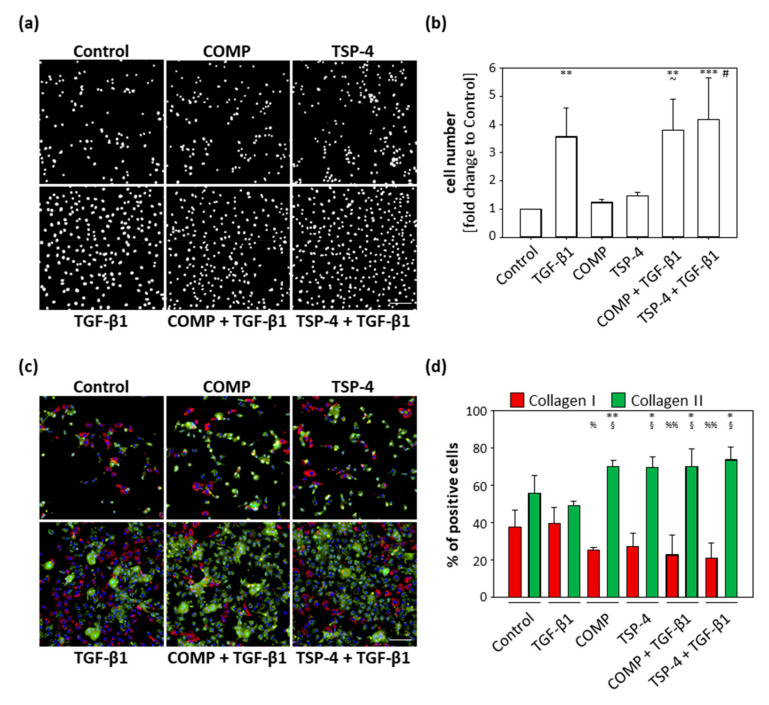
Effect of COMP and TSP-4 on chondrocyte *proliferation and dedifferentiation.* Chondrocytes were stimulated with COMP (10 µg/mL) or TSP-4 (10 µg/mL) for seven days. (**a**) Nuclei were DAPI stained at day 10. (**b**) The fold changes of the counted nuclei are represented and each bar shows the mean + SD. The significant differences between the control and all other conditions were calculated. Differences between COMP, TGF-β1 and COMP + TGF-β1 as well as TSP-4, TGF-β1 and TSP-4 + TGF-β1 were calculated. Differences to the control are indicated by an asterisk *, to COMP by waves ~ and to TSP-4 by rhombus #, as well as significance indicated by *p* ≤ 0.05 #~; *p* ≤ 0.01 **; *p* ≤ 0.001 ***. (**c**) Immunofluorescence staining of collagen I (red), collagen II (green) and nuclei (blue) at day 10. (**d**) Percentages of chondrocytes expressing collagen I and II are represented and each bar shows the mean + SD. The significant difference between collagen I and collagen II positive cells was calculated and significance indicated as *p* ≤ 0.05 *; *p* ≤ 0.01 **. Differences of collagen I and collagen II positive cells to the control were calculated and significance indicated as *p* ≤ 0.05 % or §; *p* ≤ 0.01 %%, respectively. TGF-β1 (0.5 ng/mL) served as an inducer of proliferation and collagen synthesis and unstimulated cells as a control. (*n* = 3); scale bar = 100 µm.

TGF-β1 served as an inducer of chondrocyte proliferation. COMP (*p* = 0.99) and TSP-4 (*p* = 0.86) alone had no effect on cell proliferation, and cell numbers were comparable to the untreated control (Figure 4a,b) after 10 days. TGF-β1 (*p* = 0.003) alone and in combination with COMP (*p* = 0.002) or TSP-4 (*p* < 0.001) resulted in an increased cell number. The dual treatments with TGF-β1 also increased the cell numbers compared to the equivalent single treatments with COMP (*p* = 0.013) and TSP-4 (*p* = 0.023). These data show that COMP and TSP-4 do not affect cell proliferation, either directly or by modulating the TGF-β1 induced proliferation.

The percentage of cells expressing collagen I and collagen II, respectively, was investigated by immunofluorescence double staining (Figure 4c). Collagen I and collagen II expressing cells were counted and the percentage of collagen expressing cells was calculated (Figure 4d). The percentage of collagen I and collagen II expressing cells was comparable between the control (*p* = 0.21) and TGF-β1 (*p* = 0.22) treated cultures. For all other treatments, stimulation with COMP (*p* = 0.003), TSP-4 (*p* = 0.03) or in combination with TGF-β1 (COMP: *p* = 0.05; TSP-4: *p* = 0.02) resulted in a shift towards collagen II producing cells. The percentage of unstained cells was comparable between groups, ranging from 3 to 11%. Furthermore, the number of collagen I producing cells was lower in cultures treated with COMP (*p* = 0.025) and the costimulations of TGF-β1 with COMP (*p* = 0.007) and TSP-4 (*p* = 0.004). The stimulation with TSP-4 (*p* = 0.056) showed a tendency of a reduced number of collagen I positive cells. The opposite was observed for collagen II producing cells, whose numbers increased in cultures treated with COMP (*p* = 0.033), TSP-4 (*p* = 0.042) and in the combination of TGF-β1 with COMP (*p* = 0.034) and TSP-4 (*p* = 0.010).

These results show that COMP and TSP-4 are able to suppress chondrocyte dedifferentiation in monolayer culture.

### 2.5. COMP and TSP-4 Induce the Phosphorylation of Erk1/2 While Not Affecting the Smad Pathways

COMP and TSP-4 participate in cartilage maintenance and repair by stabilizing the chondrocyte phenotype and inducing the synthesis of important ECM components. To unravel potential signaling pathways involved, the phosphorylation of Erk and Smad proteins was investigated after stimulation with COMP and TSP-4, respectively.

**Figure 5:**
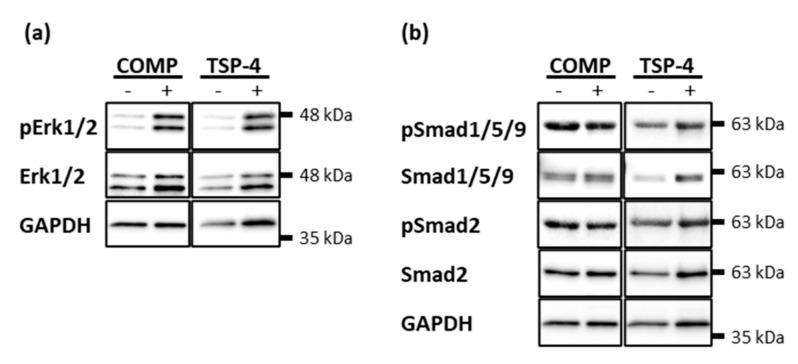
Erk and Smad phosphorylation in chondrocytes after stimulation with COMP and TSP-4. Chondrocytes were stimulated with 10 µg/mL COMP or TSP-4. Cell extracts were harvested and Smad as well as Erk proteins detected by immunoblot. (**a**) Representative immunoblots showing the detection of pErk1/2 and total Erk1/2. (**b**) Representative immunoblots showing the detection of pSmad1/5/9, Smad1/5/9, pSmad2 and Smad2. GAPDH was used as a loading control; (*n* = 4).

Chondrocytes were treated with 10 µg/mL COMP or TSP-4 for 30 min and phosphorylated Erk1/2, Smad2 and Smad1/5/9 detected by immunoblot analyses. Both COMP and TSP-4 induced the phosphorylation of Erk1/2 (Figure 5a). Even though the treatment with COMP and TSP-4 seemed to slightly increase the level of total Erk1/2, the increase in phosphorylation was much more pronounced. None of the proteins affected the phosphorylation of Smad2 or Smad1/5/9 (Figure 5b).

### 2.6. COMP and TSP-4 Can Modulate TGF-β1 Induced Erk1/2 Signaling

**Figure 6:**
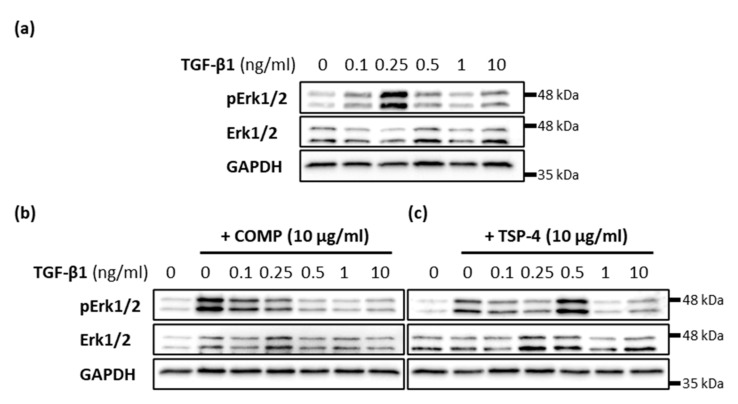
Modulation of TGF-β1-induced Erk signaling in chondrocytes. Chondrocytes were stimulated with indicated concentrations of TGF-β1 alone or in combination with COMP (10 µg/mL) or TSP-4 (10 µg/mL) before Erk1/2 proteins were detected by immunoblot. (**a**) Representative immunoblots show the concentration-dependent TGF-β1 induced phosphorylation of Erk1/2 (pErk1/2) and total Erk1/2. Representative immunoblots show the influence of COMP (**b**) and TSP-4 (**c**) on the concentration-dependent TGF-β1 induction of Erk1/2 phosphorylation. GAPDH was used as a loading control; (*n* = 4).

The effect of TGF-β1 on matrix formation was modulated by the simultaneous addition of COMP and TSP-4, respectively. Therefore, the capacity of COMP and TSP-4 to modulate TGF-β1-induced Erk signaling was investigated. The phosphorylation of Erk1/2 was investigated 30 min after stimulation with 10 µg/mL COMP or TSP-4 and different concentrations of TGF-β1 (0.1; 0.25; 0.5; 1 and 10 ng/mL). Maximal Erk1/2 phosphorylation was detected after treatment with 0.25 ng/mL TGF-β1 (Figure 6a). The additional stimulation with TSP-4 shifted this phosphorylation maximum to a TGF-β1 concentration of 0.5 ng/mL (Figure 6c). The most pronounced Erk1/2 phosphorylation with simultaneous COMP treatment was found at 0.1 ng/mL TGF-β1, and continuously decreased with increasing TGF-β1 concentrations (Figure 6b). The phosphorylation maximum at 0.1 ng/mL TGF-β1 was weaker than that of COMP alone. These results show that TSP-4 attenuates TGF-β1 induced Erk1/2 signaling, in contrast to COMP, whose capacity to induce Erk1/2 phosphorylation is suppressed by TGF-β1.

## 3. Discussion

In the present study, we show that the presence and distribution of COMP and TSP-4 are distinguishable, especially in healthy and OA cartilage, although both proteins are re-expressed during OA progression. While COMP is ubiquitously expressed in healthy cartilage, TSP-4 is hardly detectable. In OA, COMP was found to be degraded in early and re-expressed in late-stage OA [15]. In contrast, the TSP-4 level dramatically increased in an early OA stage, and was shown to be further elevated with OA severity [39]. This differential expression pattern indicates additive roles of extracellular COMP and TSP-4 in articular cartilage that may depend on their zonal distribution.

In the past, it was shown that cells in the injured articular cartilage have the potential to migrate to the damage site, leading to a repopulation of these areas [48,49]. COMP re-expression was mainly found in the upper cartilage zones, where the structural damage was most severe, while TSP-4 was predominantly expressed in the middle and deep zone, below the layer where COMP re-expression was detected. Therefore, we hypothesized that the re-expression of COMP might be an attempt to attract chondrocytes from the surrounding area to repopulate the defect site, and that interaction with COMP and TSP-4 might induce mechanisms of ECM maintenance, protection and cartilage repair. Indeed, COMP, but not TSP-4, was demonstrated to attract chondrocytes and contribute to their anchorage [50,51]. These data suggest that COMP and TSP-4 interact with differential receptors on the chondrocyte surface and are distinguishable in their functional role. Although the presence of TSP-4 is insignificant for chondrocyte migration, its rapid upregulation in injury is associated with the regulation of matrix protein synthesis and ECM remodeling [52,53,54]; both processes are highly relevant in OA. Also, induction of ECM protein synthesis seems highly likely and beneficial for COMP after mediating chondrocyte migration and attachment.

The effects of COMP-TGF-β1 and TSP-4-TGF-β1 complexes were also investigated, due to the important role of TGF-β1 in healthy cartilage, its high abundance in OA and its susceptibility to modulation by COMP after binding [23,33,47]. In our study, we showed, for the first time, that not only COMP, but also TSP-4 directly interacts with TGF-β1 (Appendix A), although TGF-β1 only induced the expression of COMP and not TSP-4 (Appendix B). These findings, together with the low expression of TSP-4 under healthy conditions, suggest a minor role in the maintenance of healthy cartilage. In addition to TGF-β1, COMP synthesis can be increased by mechanical loading, as shown in cartilage explants [55,56]. In the presence of cartilage lesions, the loading environment changes; this may lead to an altered response of chondrocytes, which deform less axially but are exposed to increased tensile strength compared to cells in an intact matrix [57,58]. Therefore, an altered loading environment in articular cartilage might also affect the expression of TSP-4 and further increase the levels of COMP. At the cellular level, we could show that both COMP and TSP-4 supported the synthesis and matrix deposition of collagen II, IX, XII and proteoglycans, while inhibiting those of collagen I and collagen X. The modulating effect of COMP has also been described before, showing that the addition of COMP or aggrecan suppresses the expression of hypertrophic genes in periosteal chondrocytes [59]. The increased expression of the main ECM components, collagen II and proteoglycans, as well as their potential to enhance TGF-β1 induced collagen II and proteoglycan synthesis, strongly suggest an involvement of both proteins in cartilage repair by restoring an ECM composition that maintains original tensile strength and elasticity [1]. Collagen fibrils in articular cartilage consist mainly of collagen II [4], and their zonal specific biomechanical, as well as structural properties are achieved by its interaction with different ECM components such as collagen IX [4,5,6], resulting in a heterogeneity of collagen II cartilage fibrils [6,7,8]. Therefore, the effect of COMP and TSP-4 on the expression of minor collagens, like collagen IX and XII, is also of great interest. While COMP and TSP-4 can both bind fibrillar collagens [40,41,42], they behave differently in terms of their capacity to bind minor collagens. In that manner, COMP was shown to interact with collagen IX [60,61] and collagen XII [43], while TSP-4 cannot directly bind to collagen IX [62], and the binding to collagen XII has not yet been investigated. Collagen XII, TSP-4 and COMP are all localized in the superficial zone in healthy cartilage, which possesses properties, such as high tensile strength to withstand the immense forces of articulation [5,8]. COMP and TSP-4 induce the synthesis of collagen XII, and may therefore increase the tensile properties in the superficial zone and prepare the underlaying tissue for increased loading. Especially in OA, affecting all cartilage layers, increased collagen XII levels may enhance the integrity of the ECM by strengthening collagen II fibrils. However, in combination with TGF-β1, this increase seems reversed, maybe due to a negative feedback loop. TGF-β1 stimulation resulted in high levels of immobilized and soluble collagen XII. These levels were much higher than those seen in COMP and TSP-4 stimulated cells. Downregulation of collagen XII might be a consequence of the binding of TGF-β1 to COMP and TSP-4, blocking relevant binding sites, e.g., on growth factor receptors, as reported for a COMP-BMP-2 complex [63]. Conspicuously, collagen XII amounts were low in conditions where collagen IX was enriched. Also, collagen IX-KO mice showed an increased amount of collagen XII distributed in areas where collagen XII is usually not expressed [62]. Both FACIT collagens share sequence homologies and are associated with collagen organization [64], indicating a compensatory role of collagen XII in the absence of collagen IX. While collagen IX is rather associated with thinner collagen fibrils, lacking decorin [4,6,7], collagen XII is a known interaction partner, suggesting the involvement in the organization of collagen fibrils with larger diameter [65] and the tensile strength of articular cartilage. COMP and TSP-4 stimulation had different effects on collagen IX expression, which was upregulated by both proteins. However, increased amounts of soluble collagen IX were only detected in the supernatant of COMP stimulated cells, indicating an impaired matrix integration, maybe due to missing binding sites. Alternatively, the increased amounts of collagen IX could form complexes with COMP in the supernatant. COMP can directly bind to collagen IX with its C-terminal domain [60], suggesting a higher affinity of collagen IX to COMP than to other binding partners in the matrix. Collagen IX is entirely integrated into the cell-associated matrix when stimulated with TSP-4. The reason for this might, therefore, be a lower expression capacity or the different binding properties to collagen IX in comparison to COMP [62], excluding the possibility of a complex formation. Furthermore, the stimulation with COMP and TSP-4 resulted in a partially reduced amount of collagen I, and led to a shift in direction collagen II positive cells. Several studies have reported that prolonged culture of chondrocytes in monolayer lead to dedifferentiation, characterized by an increase of collagen I expression and, at the same time, a loss of cartilage-specific markers, like collagen II, collagen IX and, in particular, COMP [35,66]. Therefore, it is tempting to speculate that stimulation with COMP would counteract this dedifferentiation process. Indeed, our observations seem to confirm this hypothesis of chondrocyte phenotype stabilization after stimulation with COMP and also TSP-4, though to a lesser extent. However, the loss of collagen IX, visible in TGF-β1 stimulated cultures, could not be reversed by a simultaneous treatment with COMP or TSP-4, demonstrating a dominant effect of TGF-β1 on collagen IX expression and eventually also on the chondrocyte phenotype. A beneficial effect of COMP in cartilage regeneration was demonstrated by Wang C, et al. [67], showing that an overexpression of COMP promoted chondrogenic differentiation of bone-marrow derived stem cells, resulting in an increased formation of articular cartilage. Therefore, COMP and TSP-4 might be promising factors for clinical applications, e.g., in maintaining the chondrocyte phenotype during in vitro expansion for autologous chondrocyte implantation (ACI) or incorporated into scaffolds, improving cell anchorage, protecting chondrocytes phenotype and inducing the production of proteins, essential for ECM properties.

After having demonstrated potential functional roles of COMP and TSP-4 in articular cartilage, we aimed to gain more insight into the involved signaling pathways. Chondrocytes in mature cartilage facilitate the maintenance of the ECM in a low turnover state [17], which requires a delicate balance of catabolic and anabolic processes, which are regulated by, among other mechanisms, growth factors like TGF-β1 [23,24,25,26]. The most relevant pathways to maintain cartilage homeostasis are the Smad (small mothers against decapentaplegic homologs) and Erk1/2 signaling [26]. The counteracting Smad2/3 and Smad1/5/9 have different Smad-binding motifs in the DNA sequence, resulting in the downstream transcription of differential genes [26,68]. TGF-β1 typically induce Smad2/3 signaling, promoting matrix maintenance by inhibiting chondrocyte hypertrophy [29,30] and inducing the expression of matrix proteins like aggrecan and collagen II [24], similar to Erk1/2 [24,32]. Smad1/5/9 phosphorylation results in the opposite, i.e., the expression of collagen X and MMP-13, causing chondrocyte hypertrophy and matrix degradation [31]. In OA, reactivated chondrocytes fail to regulate the metabolism properly, leading to an imbalance of anabolic and catabolic processes. For presently unknown reasons, chondrocytes favor Smad1/5/9 and switch from an anabolic to a catabolic Erk1/2 signaling, in OA, leading to an unstable chondrocyte phenotype and ECM degradation [69,70,71]. The capacity of COMP and TSP-4 to induce the Erk1/2 signaling pathway has already been demonstrated in other cell types, including primary hepatic stellate cells [72] and cardiomyocytes [73], respectively. Thereby, the Erk phosphorylation could be induced via the CD36 receptor [72], a receptor naturally expressed on healthy chondrocytes and even increasing in the pathology of OA [74]. In our study, we showed that COMP and TSP-4 could also induce the phosphorylation of Erk1/2 in articular chondrocytes, while none of the proteins had an effect on Smad1/5/9 or Smad2/3 signaling. Interestingly, it has been shown earlier that other ECM components, like e.g. collagen II, were able to suppress hypertrophy in articular chondrocytes via the Erk1/2 signaling pathway [32]. The similar effects of COMP and TSP-4 on protein synthesis and cell-signaling indicate that the upregulation of proteoglycan and collagen II, as well as the downregulation of collagen X, might be mediated via Erk1/2 signaling, while migration is most likely performed by an Erk1/2-independent mechanism. Nonetheless, future studies are necessary to confirm the role of Erk1/2, as well as to identify the responsible receptors.

The present study was limited by the availability of healthy human articular cartilage samples. The distribution and expression of COMP and TSP-4 was investigated in human osteoarthritic and healthy cartilage samples. Thereby, we could show that the ECM composition had already been altered in an early OA stage (G1), i.e., at which obvious morphological defects were not yet detectable. Therefore, healthy pig cartilage was used to study the effects of COMP and TSP-4 on chondrocytes in vitro. The effect of COMP and TSP-4 on OA chondrocytes has to be investigated in more detail in future experiments.

**Figure 7:**
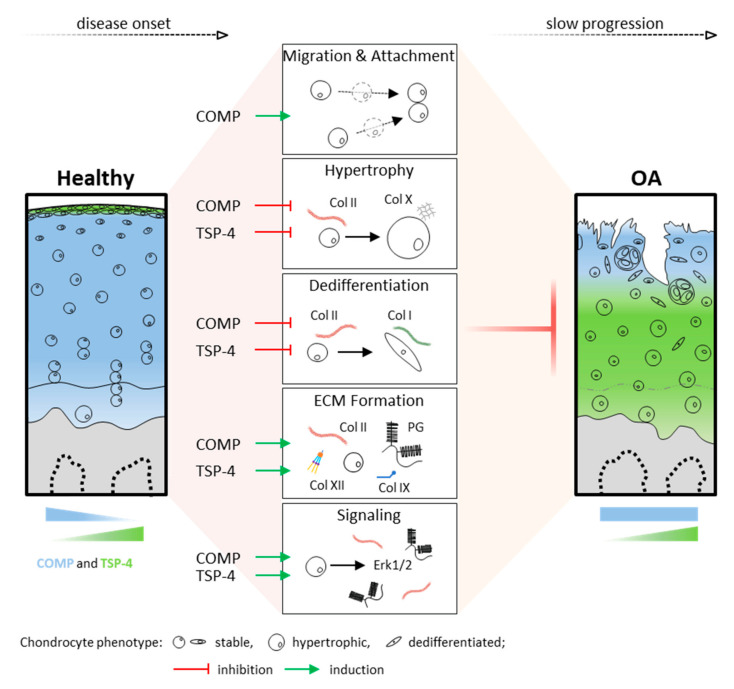
Functional role and distribution of COMP and TSP-4 in articular cartilage. Schematic representation of COMP and TSP-4 distribution, as well as protein levels in healthy and OA cartilage. Areas of COMP and TSP-4 expression are indicated in blue and green, respectively. Their impact on cartilage and OA relevant processes is depicted in the middle. Green arrows indicate an induction, while red T arrows mean inhibition. PG = proteoglycan and Col = collagen.

In summary, these data demonstrate that chondrocytes in articular cartilage can activate mechanisms to protect and repair the ECM in injury and disease. COMP and TSP-4, distributed in different cartilage zones, might contribute to the integrity and repair of the ECM by promoting chondrocyte migration and ECM protein synthesis, as well as the stabilization of the chondrocyte phenotype (Figure 7). Their involvement in direct and indirect cell signaling emphasizes the complexity of cell-matrix interactions and intracellular events in degenerative processes. Overall, OA leads to cartilage degradation, despite the protective effects of COMP and TSP-4. However, these repair processes might help to slow the progression of OA, and thus pave the way for new treatment strategies.

## 4. Materials and Methods

### 4.1. Collection and Scoring of Human Osteochondral Cylinders

Adult, human, anonymized cartilage samples were obtained from seven OA patients undergoing endoprosthetic knee replacement surgery at the Department of Orthopaedics (Friedrichsheim), University Hospital Frankfurt, Goethe University, and visually scored as described before [39]. Intact cartilage areas with a smooth and shiny surface were scored as grade 1 (G1), cartilage areas with superficial fissures and a rough surface were scored as grade 2, and areas with deeper fissures and/or exposure of subchondral bone as grade 3 to 4 (G3/4). Two non-OA cartilage sections staining were a gift from Gerjo van Osch (Erasmus MC University Rotterdam, Rotterdam, The Netherlands) and used as healthy controls.

### 4.2. Chondrocyte Isolation and Culture from Pig Articular Cartilage

Pig legs were obtained from the animal house at Goethe University Frankfurt, Frankfurt/Main, Germany from among animals sacrificed within the scope of other scientific projects. Legs from female, healthy pigs (three to six months old) were received immediately after sacrificing. Pig legs were processed to expose the knee and the knee joint was opened to scrape off the articular cartilage from the femoral condyles under sterile conditions. The cartilage was washed with phosphate-buffered saline (PBS; Thermo Fisher Scientific, Waltham, MA, USA) and cut into small pieces (2–3 mm^3^) to isolate chondrocytes. The cartilage pieces of pig knee joints were weighted, transferred into a sterile tube and digested with 0.2% pronase (Roche Diagnostics, Mannheim, Germany) in DMEM/F12 medium containing 5% FBS and 5% pen/strep (all from Gibco, Karlsruhe, Germany) for 2 h at 37 °C with the agitation of 60 rpm. After incubation, cells and cartilage pieces were pelleted by centrifugation at 300× *g*, 5 min at RT and the supernatant decanted. Cells and cartilage pieces were washed 3× with PBS and digested with 200 U/mL collagenase type II (Biochrom, Berlin, Germany) solution in DMEM/F12 medium (containing 5% FBS and 5% pen/strep) overnight at 37 °C with the agitation of 60 rpm. The chondrocyte suspension was filtered through a 70 μm nylon cell strainer and centrifuged at 300 x g for 5 min at RT. The pelleted cells were resuspended, counted with a Neubauer chamber and seeded either in DMEM/F12 containing 5% FBS or resuspended in medium containing 0.1% bovine serum albumin (BSA; PanReac, AppliChem, Darmstadt, Gemany) for direct use in the migration assay. All cells were cultured at 37 °C, 5% CO_2_ and 20% O_2_ in a CO_2_-incubator.

To analyze the production of matrix proteins and matrix formation via immunofluorescence staining and immunoblot analysis, 40,000 cells in DMEM/F12 containing 5% FBS were seeded in each chamber of a Nunc^TM^ Lab-Tek^TM^ II four-well chamber slide (Thermo Fisher Scientific, Waltham, MA, USA). Chondrocytes used for signaling experiments were seeded in a density of 300,000 cells in DMEM/F12 containing 5% FBS per well of a six-well plate. The cells were allowed to attach to the bottom overnight. The following day, the cells were washed twice with PBS and starved in basic medium (DMEM/F12 containing 0.1% BSA and 25 µg/mL ascorbic acid (Sigma-Aldrich, St. Louis, MO, USA)) for 24 h. Then, the cells were stimulated with recombinant proteins. Different concentrations of TGF-β1 (R&D Systems, Minneapolis, MN, USA) were used, depending on the downstream assay. In all assays, recombinantly expressed and purified COMP and TSP-4 were used in a concentration of 10 µg/mL. The chondrocytes were treated with TGF-β1, COMP or TSP-4 alone and in the combination of TGF-β1 with COMP or TSP-4 in basic medium. When cells were stimulated in the combination of TGF-β1 with COMP or TSP-4, the two components were mixed in a tube and pre-incubated for 20 min at RT, before applying to the cells.

Chondrocytes seeded in chamber slides were stimulated with 0.5 ng/mL TGF-β1, 10 μg/mL COMP, 10 μg/mL TSP-4 and in the combination of TGF-β1 with COMP or TSP-4 for a period of seven days. The chondrocytes were first stimulated with recombinant protein in the basic medium for four days. The medium was changed at day 4 and recombinant proteins applied in a basic medium containing 5% FBS. At day 7, the medium was replaced by a basic medium containing only 5% FBS and chondrocytes cultured until day 10. The supernatants were collected after each medium change and stored at –20 °C.

For immunoblot analyses, chondrocytes were stimulated with 0.1 ng/mL, 0.25 ng/mL, 0.5 ng/mL, 1 ng/mL and 10 ng/mL TGF-β1, 10 μg/mL COMP, 10 μg/mL TSP 4 or a combination of all TGF-β1 concentrations with COMP or TSP-4 for 30 min at 37 °C.

### 4.3. Expression and Purification of Recombinant Proteins

Recombinant TSP-4 and COMP were produced as described before [75,76]. Shortly, HEK-293 EBNA cells were transfected with the pCEP-Pu V162 expression vector (generated by Prof. Manuel Koch, University of Cologne) carrying the sequence of full-length rat COMP and TSP-4 and cultured in DMEM/F12 medium containing 1% FBS for a period of five days. The cell culture supernatant was collected daily and cleared by centrifugation. The recombinant proteins were purified via the Strep-Tag affinity chromatography column (IBA, Goettingen, Germany) at day five. The protein concentration was measured with the Qubit fluorometer and protein fractions stored at –20 °C until use.

### 4.4. (Immuno)histological and Immunofluorescence Staining of Cartilage Samples and Chondrocytes

Osteochondral cylinders were generated and processed as described before [39]. Samples were fixed in 4% paraformaldehyde (Sigma-Aldrich, St. Louis, MO, USA) in PBS, pH = 7.4, overnight at 4 °C. After decalcification in 10% ethylenediaminetetraacetic acid (EDTA; VWR, Osterode am Harz, Germany), samples were embedded in paraffin. Then, 5 µm sections were generated, deparaffinized and rehydrated prior to antigen retrieval. Tissue sections were treated with 250 U hyaluronidase (Sigma-Aldrich) in PBS (pH = 5) before TSP-4 staining and with 20 μg/mL proteinase K (Qiagen, Hilden, Germany) in proteinase K buffer (10 mM NaCl, 50 mM Tris-base, 10 mM EDTA in ddH_2_O, pH 7.4) before COMP staining, for 15 min at 37 °C. Endogenous peroxidase activity was blocked with 0.3% H_2_O_2_ (Carl Roth, Karlsruhe, Germany) in dH_2_O for 10 min at RT and unspecific binding blocked with 2.5% normal horse serum (included in the ImmPRESS^TM^ HRP reagent kit, Vector Laboratories, Burlingame, CA, USA) for 20 min at RT. Primary antibodies (Table 1) were diluted in 1% BSA and the tissue sections incubated at 4 °C overnight. The primary antibodies were detected with the ImmPRESS^TM^ (peroxidase) polymer anti-rabbit IgG reagent (included in the ImmPRESS^TM^ HRP Reagent Kit, Vector Laboratories, Burlingame, CA, USA) at RT for 30 min. The AEC-2-component kit (DCS, Hamburg, Germany) was used according to the manufacturer’s instructions to visualize the secondary antibody. Negative control staining without addition of the primary antibodies were carried out to exclude unspecific binding of the secondary antibody (data not shown).

Cultured chondrocytes in chamber slides were fixed with Shandon™ zinc formal-fixx™ (Thermo Fisher Scientific, Waltham, MA, USA) for 20 min at RT, washed 3x with PBS. To stain proteoglycans cells were incubated with 0.1% Safranin-O (Carl Roth, Karlsruhe, Germany) at RT for 15 min. Before antibody staining, cells were permeabilized with 0.3% Triton X-100 (Merck, Darmstadt, Germany) in PBS for 10 min at RT. Unspecific binding sites were blocked with blocking buffer (1% BSA and 1% goat serum (Abcam, Cambridge, UK) in PBS) for 1 h at RT. Cells were incubated with the primary antibodies (Table 1) diluted in 1% BSA in PBS at 4 °C overnight. After three washing steps with PBS-T (PBS containing 0.1% Tween-20 (Carl Roth, Karlsruhe, Germany)), cells were incubated with the corresponding secondary antibodies (Table 2) diluted in 1% BSA in PBS at RT in the dark for 1.5 h. The cells were washed twice with PBS-T and once with PBS before mounting with the DAPI fluoroshield mounting medium (Abcam, Cambridge, UK). DAPI was visualized at a wavelength of 461 nm, Alexa Fluor 488 at 516 nm and Alexa Fluor 594 at 617 nm with a fluorescence and Safranin-O with a light microscope (Nikon, Tokyo, Japan).

### 4.5. Protein Extraction and Analysis

Proteins from human cartilage samples were extracted as described previously [39]. Briefly, cartilage from areas showing different severity grades were scraped off and cut into pieces (1–3 mm^3^), and proteins were extracted with an extraction buffer (4 M guanidine hydrochloride, 50 mM Tris, 10 mM EDTA (pH = 7.4)) overnight at 4 °C. After precipitating the proteins with 96% ethanol for 24 h at –20 °C, the protein pellet was washed and resuspended in Laemmli buffer (250 mM Tris-HCl, pH 6.8, 40% glycerol, 0.04% bromophenol blue, 8% SDS).

Cells in monolayer culture were washed with PBS and cell lysates collected by scraping the cells in 1x Laemmli buffer containing 40% β-mercaptoethanol (Sigma-Aldrich, St. Louis, MO, USA) and phosphoSafe^TM^ (Merck, Darmstadt, Germany). Cell culture supernatants were mixed with Laemmli buffer with or without β-mercaptoethanol.

For quantification, proteins were separated by SDS-PAGE using 5%, 8% or 10% polyacrylamide gels as previously described [39]. The separation was carried out using a Mini-PROTEAN^®^ Tetra-cell system (Bio-Rad, Munich, Germany) at 150 V and equal loading was demonstrated by PageBlue™ (Thermo Fisher Scientific, Waltham, MA, USA) or Roti^®^blue (Carl Roth, Karlsruhe, Germany) staining of total proteins. After electrophoresis, proteins were transferred onto a 0.45µm polyvinylidene fluoride (PVDF) membrane (GE Healthcare, Freiburg, Germany) using the mini Trans-blot^®^ electrophoretic transfer cell (Bio-Rad, Munich, Germany) and unspecific binding sites blocked in 10% skim milk (COMP and TSP-4) or 5% BSA (signaling proteins and collagens) at RT for 1 h with gentle agitation. Membranes were incubated with the corresponding antibodies listed in Table 1 at 4 °C overnight. After washing, membranes were incubated with the corresponding secondary antibodies (Table 2) for 1 h at RT and the proteins visualized by using a mixture of a homemade ECL solution (0.1 M Tris-HCl, pH = 8.5; 225 mM *p*-coumaric acid and 1.25 mM luminol) with 3% H_2_O_2_. The protein signals were analyzed by the Chemi Doc^TM^ XRS+ (Bio-Rad, Munich, Germany) molecular imager and the ImageLab^TM^ software (http://www.bio-rad.com/de-de/product/image-lab-software) (accessed on 1 January 2021). The band intensities were quantified with the ImageJ version 1.5 software (http://imagej.nih.gov/ij) (accessed on 1 January 2021).

### 4.6. Migration Assay

The transwell system with ThinCert^TM^ cell culture inserts containing a polyethylene terephthalate (PET) membrane with a pore size of 8 µm was used in combination with 24-well plates to investigate the migration of primary pig chondrocytes. First, 400 µL DMEM/F12 containing 0.1% BSA with either 10 µg/mL COMP or 10 µg/mL TSP-4 were added to the lower compartment of the transwell system. As a positive control, chondrocytes were attracted with 10 ng/mL platelet-derived growth factor-BB (PDGF-BB) (Miltenyi Biotec, Bergisch Gladbach, Germany). As a negative control, the plenty medium was added in the lower compartment. Chondrocytes were directly used after isolation from knee cartilage and washed twice with PBS before use in migration assays. Then, 50,000 cells in 200 µL medium were transferred to the upper compartment of the inserts. After 10 h incubation at 37 °C, 5% CO_2_ and 20% O_2_, the cells within and on the lower side of the PET membrane were fixed with Shandon™ zinc formal-fixx™ for 20 min at RT. Nonmigrated chondrocytes on the upper side of the PET membrane were removed using a cotton swab. After a brief rinse in dH_2_O, the nuclei were stained with DAPI (Sigma-Aldrich, St. Louis, MO, USA) and imaged with a fluorescent microscope at a wavelength of 461 nm. Five pictures from different areas of the membrane were taken and the number of cells counted with ImageJ. The fold change of the number of cells which had migrated toward COMP, TSP-4 and the positive control were calculated based on the number of cells which had migrated toward the negative control. All technical replicates were performed at least in duplicate.

### 4.7. Attachememt Assay

Wells of a 96-well plate were coated with 10 µg/mL COMP and TSP-4 in PBS. As a negative control 100 mg/mL BSA and as a positive control 10 µg/mL fibronectin (R&D Systems, Minneapolis, MN, USA), were used. The recombinant protein, in the indicated concentration, were added to each well and incubated overnight at 4 °C. The following day, the wells were washed twice for 5 min with PBS and once for 5 min with DMEM/F12 containing 0.1% BSA. After blocking with 1% BSA for 3 h at RT, chondrocytes were added at a concentration of 50,000 cells in DMEM/F12 containing 0.1% BSA. Chondrocytes were incubated for 1 h at 37 °C and 5% CO_2_ in a CO_2_-incubator. Following the incubation, the supernatant was carefully removed and the wells briefly washed twice with PBS. Chondrocytes were fixed with Shandon™ zinc formal-fixx™ for 20 min at RT. Cells were washed three times for 5 min each with PBS and stained with 0.1% crystal-violet for 30 min at RT. The cells were washed three times for 5 min each with ddH_2_O and imaged with a light microscope. The number of cells was counted and the fold change to the negative control calculated. All technical replicates were performed in triplicates.

### 4.8. Surface Plasmon Resonance Spectroscopy

The interaction between proteins was measured with the Biacore 2000 (Biacore AB, Uppsala, Sweden). All experiments were performed at 25 °C and with a CM5 sensor chip coupled with 1500 U TGF-β1. As a reference, a flow cell without an immobilized ligand was used. A 1:2 dilution series from a concentration of 160 nm to 0 nm of the analytes, COMP and TSP-4, were prepared in a running buffer (Biacore AB, Uppsala, Sweden). The system was equilibrated at a flow rate of 30 µl/min with the running buffer, and the experiment started when a stable baseline was reached. The injection of the analyte was started and data were collected for kinetic analysis. One analyte concentration per cycle was injected, starting with the lowest concentration. The chip was regenerated after each cycle with 2 M NaCl.

The BIAevaluation software 3.0 (Biacore AB, Uppsala, Sweden) was used to create a sensorgram, showing the response against the time. By doing a multicycle kinetic, several analyte concentrations were induced in separate cycles, resulting in multiple curves in the sensorgram. By fitting the curves to the mathematical 1:1 model, the association rate (ka), dissociation rate (kd) and the dissociation constant (K_d_) was evaluated.

### 4.9. Statistical Analysis

The SigmaPlot version 13.0 software (Systat Software, Inc., San Jose, CN, USA) was used for statistical analyses. Differences between groups were evaluated by the paired *t*-test, One Way ANOVA or the Friedman test with either the Tukey, Student-Newman-Keuls Method or Dunnett’s post hoc test. Correlations between groups were analyzed by using the Spearman rank test (r). A *p*-value < 0.05 was considered as significant difference (*p* < 0.05 *; *p* ≤ 0.01 **; *p* ≤ 0.001 ***).

## Figures and Tables

**Table 1:** List of primary antibodies.

Target	Host	Dilution	Source or Supplier
Collagen I	rabbit	1:200 (IF), 1:1000 (IB)	Abcam (ab34710)
Collagen II	mouse	1:200 (IF), 1:1000 (IB)	Merck (CP18)
Collagen IX	guinea pig	1:200 (IF), 1:1000 (IB)	[77]
Collagen X	mouse	1:50 (IF), 1:100 (IB)	Mengjie Zhou (University of Cologne)
Collagen XII	rabbit	1:200 (IF), 1:1000 (IB)	Manuel Koch (University of Cologne)
COMP	rabbit	1:100 (IF), 1:500 (IHC), 1:1000 (IB)	[78]
TSP-4	rabbit	1:100 (IF), 1:500 (IHC)	[39]
TSP-4	guinea pig	1:1000 (IB)	[40,79]
Smad2	rabbit	1:1000 (IB)	Cell Signaling (5339)
pSmad2	rabbit	1:2000 (IB)	Cell Signaling (3108)
Smad1/5/9	rabbit	1:500 (IB)	Santa Cruz (sc-6031-R)
pSmad1/5/9	rabbit	1:1000 (IB)	Cell Signaling (13820)
Erk1/2	mouse	1:2500 (IB)	Cell Signaling (9107)
pErk1/2	rabbit	1:2000 (IB)	Cell Signaling (4370)
GAPDH	mouse	1:2000 (IB)	Thermo Fisher Scientific (MA5-15738)

**Table 2:** List of conjugated antibodies.

Target	Host	Conjugated	Dilution	Reference or Producer
Rabbit IgG	goat	HRP	1:1000 (IB, IHC)	Agilent (P0448)
Guinea pig IgG	rabbit	HRP	1:1000 (IB, IHC)	Agilent (P014102-2)
Mouse IgG	goat	HRP	1:1000 (IB, IHC)	Agilent (P0447)
Rabbit IgG	goat	Alexa Fluor 594	1:500 (IF)	Invitrogen (A-11037)
Guinea pig IgG	goat	Alexa Fluor 488	1:500 (IF)	Invitrogen (A-11073)
Mouse IgG	goat	Alexa Fluor 488	1:500 (IF)	Invitrogen (A-11029)

## Data Availability

The data presented in this study are available on request from the corresponding author.

## Abbreviations

BMP	Bone morphogenetic protein
BSA	Bovine serum albumin
Col	Collagen
COMP	Cartilage oligomeric matrix protein
DAPI	4′,6-diamidino-2-phenylindole
DMEM	Dulbecco’s Modified Eagle’s Medium
EBNA	Epstein-Barr virus nuclear antigen
ECM	Extracellular matrix
EDTA	Ethylenediaminetetraacetic acid
Erk	Extracellular-signal regulated kinases
EtOH	Ethanol
F	Fraction
FACIT	Fibril-associated collagens with interrupted triple helices
FBS	Fetal bovine serum
G	Grade
GAPDH	Glycerinaldehyd-3-phosphat-dehydrogenase
HC	Healthy control
HEK cells	Human embryonic kidney cells
HRP	Horseradish peroxidase
IB	Immunoblot
IF	Immunofluorescence
IHC	Immunohistochemistry
ka	Association rate
kd	Dissociation rate
k_D_	Dissociation constant
kDa	Kilo Dalton
LC	Loading control
OA	Osteoarthritis
PBS	Phosphate-buffered saline
PDGF	Platelet-derived growth factor
Pen/strep	Penicillin/streptomycin
PET	Polyethylene terephthalate
PG	Proteoglycan
PVDF	Polyvinylidene fluoride
Rec	Recombinant
rpm	Revolutions per minute
RT	Room temperature
RU	Response Units
SDS-PAGE	Sodium dodecyl sulfate polyacrylamide gel electrophoresis
Smad	Small mothers against decapentaplegic homologs
TGF-β1	Transforming growth factor-beta 1
TSP-4	Thrombospondin-4

## Appendix A. Both, COMP and TSP-4 Interact with TGF-β1

COMP and TSP-4 had similar capacities to contribute to the ECM formation and could further modulate TGF-β1 induced Erk1/2 signaling. A direct interaction of COMP with TGF-β1 has been described earlier. This interaction was shown to modulate the bioactivity of TGF-β1 [47]. However, it was unclear if TSP-4 also directly interacts with TGF-β1. Therefore, the interaction of both proteins with TGF-β1 was characterized in surface plasmon resonance measurements using the Biacore system. The protein-protein interaction profiles show that both COMP (Figure A1a) and TSP-4 (Figure A1b) interact with TGF-β1. According to the calculated K_d_ value, the affinity of TSP-4 for TGF-β1 seems to be slightly stronger than that of COMP. However, due to the low dissociation rate of COMP, a direct comparison is difficult (Figure A1c).

## Appendix B. TGF-Β1 Promotes the Synthesis and Deposition of COMP but Not TSP-4

Previously, it was shown that COMP and TSP-4 could directly bind to TGF-β1 and modulate its bioactivity. Further, Motaung SC, et al. [80] showed that TGF-β1 could also induce the synthesis of COMP. To investigate if also TSP-4 is induced after TGF-β1 treatment, primary chondrocytes were stimulated with 0.5 ng/mL TGF-β1 for seven days in monolayer culture and the matrix deposition of COMP and TSP-4 stained with specific antibodies at day 10. TGF-β1 could induce the synthesis and matrix deposition of COMP but not TSP-4 (Figure A2a). Soluble COMP could be detected in the supernatant of controls and to a larger extend in the supernatant of TGF-β1 stimulated cells, although in none of the cultures soluble TSP-4 could be detected (Figure A2b).
